# An Essay on the Tongue, in Functional Derangement of the Stomach and Bowels

**Published:** 1844-07

**Authors:** 


ART. VI.—*An Essay on the Tongue, in Functional Derangement of the Stomach and Bowels.* By E. WILLIAMS, M.B. — *London,* 1843. 8vo, pp. 120.

IN this small volume Dr. Williams has endeavoured to trace the connexion between functional derangement of the stomach and bowels, and the appearance of the tongue. He has noted a large number of cases, placed them in tabular order, and drawn inferences and deductions. He has shown much industry, and no wish to generalize too hastily. There is, however, a want of clearness in his style so that his meaning is obscure. In the first page for instance: "An undue anxiety has been evinced to view this organ (the tongue) as an invariable monitor of functional derangement, or membranous disunion." What he intends by "membranous disunion," we cannot in the least imagine. Although fearing from the first that an author who wrote so loosely, would be found deficient in the first requisites of a teacher, we read on, as in duty bound; and were sorry to find throughout, something of the same difficulty at arriving at a distinct conception of many of the inferences and deductions. On this account we are prevented from giving our own impressions of them, as we do not feel certain it is the correct one. We trust that Dr. Williams will pursue the subject which is an important one, and digest his matter more completely. The want of clearness may be partly owing to the author being an unpractised writer, partly to the difficulty of the subject itself; but it also usually happens that where there is obscurity in expression, there is obscurity in thought.

